# The Role of Data-Independent Acquisition for Glycoproteomics

**DOI:** 10.1074/mcp.R120.002204

**Published:** 2021-01-07

**Authors:** Zilu Ye, Sergey Y. Vakhrushev

**Affiliations:** Departments of Cellular and Molecular Medicine, Faculty of Health Sciences, Copenhagen Center for Glycomics, University of Copenhagen, Copenhagen N, Denmark

**Keywords:** data-independent acquisition, post-translational modification, glycosylation, glycoproteomics, DDA, data-dependent acquisition, DIA, data-independent acquisition, ETD, electron transfer dissociation, FDR, false discovery rate, HCD, higher-energy collisional dissociation, PNGase F, peptide-N-glycosidase F, PTMs, post-translational modifications, RT, retention time, SA, sialic acid, SRM, selected reaction monitoring, SWAT-MS, sequential window acquisition of targeted fragment ions, SWATH-MS, sequential window acquisition of all theoretical mass spectra

## Abstract

Data-independent acquisition (DIA) is now an emerging method in bottom–up proteomics and capable of achieving deep proteome coverage and accurate label-free quantification. However, for post-translational modifications, such as glycosylation, DIA methodology is still in the early stage of development. The full characterization of glycoproteins requires site-specific glycan identification as well as subsequent quantification of glycan structures at each site. The tremendous complexity of glycosylation represents a significant analytical challenge in glycoproteomics. This review focuses on the development and perspectives of DIA methodology for N- and O-linked glycoproteomics and posits that DIA-based glycoproteomics could be a method of choice to address some of the challenging aspects of glycoproteomics. First, the current challenges in glycoproteomics and the basic principles of DIA are briefly introduced. DIA-based glycoproteomics is then summarized and described into four aspects based on the actual samples. Finally, we discussed the important challenges and future perspectives in the field. We believe that DIA can significantly facilitate glycoproteomic studies and contribute to the development of future advanced tools and approaches in the field of glycoproteomics.

## Highlights

• Protein glycosylation and challenges in glycoproteomics.
• Data-independent acquisition for deglycosylated and intact N-linked glycopeptides.
• Unbiased screening of oxonium ions from all glycopeptide precursors.
• Glyco–data-independent acquisition on mucin-type O-glycopeptides.

## Graphical Abstract

**Figure:**
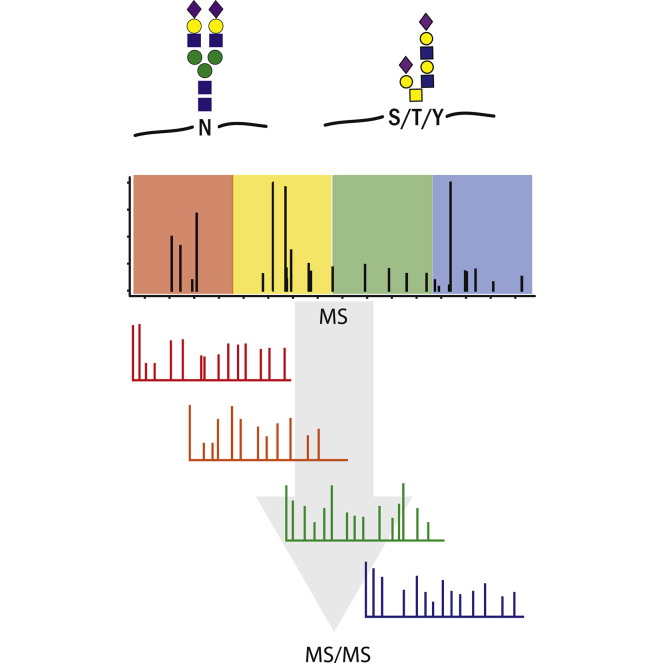

## In Brief

As a highly abundant and diverse post-translational modification, protein glycosylation is challenging to characterize in various approaches including MS. In MS-based proteomics, data-independent acquisition (DIA) has been advanced rapidly and showed outstanding analytical performances. DIA now started to be applied in different facets of glycoproteomics, including deglycosylated and intact N-linked and O-linked glycopeptides, and screening of oxonium ions. We summarized current applications of DIA in glycoproteomics and discussed its limitations and perspectives.

### N- and O-Linked Glycosylation

**Fig. 1:**
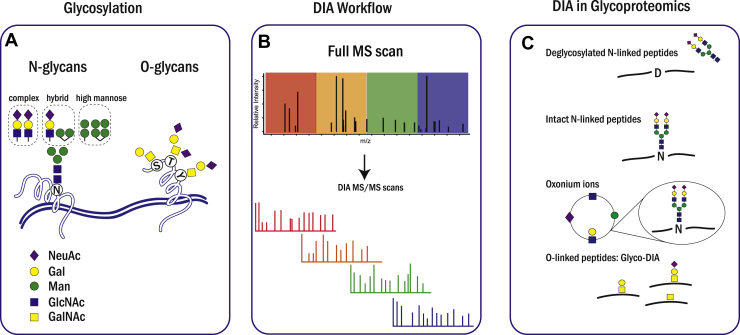
***A*, representative glycan structures on N- and O-glycosites.***B*, schematic depiction of DIA workflow. In DIA mode, a full scan is first acquired, followed by multiple MS/MS scans from all precursors in predefined consecutive mass-to-charge windows. *C*, applications of DIA in glycoproteomics. DIA-based glycoproteomics can be classified into following categories: fully or partially deglycosylated N-linked peptides, intact N-linked glycopeptides, and oxonium ions from glycopeptides and O-linked glycopeptides. The cartoons represent the peptide precursors that are analyzed. DIA, data-independent acquisition.

Protein glycosylation plays a key role in various biological processes and is one of the most abundant and diverse post-translational modifications (PTMs). Glycoproteins are a class of proteins decorated with glycans, most commonly *via* N- and O-glycosidic linkages to the side chains of the acceptor amino acid residues (1). In all eukaryotic cells, N-glycosylation processing starts with the synthesis of dolicho-linked precursor oligosaccharides (dolichol-GlcNAc2–Man9–Glc3), and it is further transferred to the nascent protein. These steps and initial trimming of the precursor molecules occur in the endoplasmic reticulum and further glycan processing continues in the Golgi apparatus. Final N-glycan structures share a common pentasaccharide core (Man_3_GlcNAc_2_) and can be extended and classified into three different subtypes: high-mannose, complex, and hybrid subtypes (2). In contrast to N- glycosylation, GalNAc-type O-glycosylation is initiated in the early Golgi apparatus by the addition of a GalNAc residue to the oxygen atom of selected Ser or Thr (in rare cases, Tyr) residues (Fig. 1*A*) (3, 4).

This initial transfer of a GalNAc residue is catalyzed by a large family of up to 20 polypeptide GalNAc-transferases (GalNAc-Ts), which are differentially expressed and show distinct but partially overlapping substrate specificities (5). The resulting most immature GalNAc-type O-glycan consists of only one GalNAc residue and is designated as the Tn antigen (GalNAc-Ser/Thr) (6). The Tn antigen is uncommon in normal mammalian glycoproteins but is often highly expressed in tumor, suggesting that the extension of O-glycans is blocked in some cancer cells (7). The addition of sialic acid (SA) to GalNAc of the Tn antigen forms the STn structure, which is commonly found in advanced tumors (7). GalNAc-type O-glycans have four major core structures (cores 1–4), and each core structure can be further extended with linear or branched chains. The immature Tn epitope is extended to generate core 1 or core 3 O-glycans. Core 1 O-glycans are formed by the addition of β1-3Gal by the T-synthase (C1GALT1) (8) and represent the most common type of O-GalNAc glycans. Core 1 glycans are often capped with SA by ST3Gal- and ST6Gal-sialyltansferases, forming monosialylated and disialylated core 1 structures, respectively (9). β1-6GlcNAc is added to core 1 O-glycans by core 2 β1-6 *N*-acetylglucosaminyltransferases 1, 2, and 3 (C2GnT-1 and GCNT1) to produce core 2 O-glycans (10). β1-3GlcNAc is added to the immature Tn epitope by core 3 synthase (C3GnT/β3GnT6) to form core 3 O-glycans (9). The M-type GCNT3 can add β1-6GlcNAc onto core 3 O-glycans to generate core 4 O-glycans (11).

Glycosylation, together with other PTMs, provides a vast spatial expansion of the proteome and adds multiple layers of complexity to the interactome. Glycoproteome identification is a multimodal process that includes peptide sequence determination, localization of glycosites on each glycoprotein, and site occupancy evaluation with the identification of all possible glycoforms on each glycosite. The complexity, stoichiometry, and heterogeneity imposed by PTMs provide further challenges for MS-based analysis for them. In principle, MS approaches are currently able to address some of the aforementioned issues individually, but they still cannot comprehensively address the general characterization of glycoproteomes, and their integration into a single protocol remain a major challenge.

### Common Challenges in Glycoproteomics

The development of approaches based on LC–MS/MS significantly facilitated high-throughput peptide sequencing, and they are currently the methods of choice for proteomic and PTM-omic studies (12). Data-dependent acquisition (DDA) bottom–up proteomics, where individual precursor ions are sequentially isolated in a narrow *m*/*z* window and fragmented, is a widespread discovery strategy in shotgun proteomics today. A major bottleneck of the DDA approach is that it is impossible to select all detected precursor ions for fragmentation in complex samples, resulting in the semistochastic identifications. In DIA-based methodology, precursor ions are isolated in a wide *m*/*z* range (usually 10–20 Th) and simultaneously cofragmented making complex chimeric MS/MS spectrum (Fig. 1*B*). This should resolve the issue of stochastic sampling in DDA mode and increase the detection sensitivity level and quantification accuracy (13).

Another challenge of the bottom–up glycoproteomics comes from the fact that glycopeptides are normally isolated together with the vast amount of nonglycosylated peptides. Because of such high complexity, glycopeptide ions could either be low abundant or largely suppressed, requiring special enrichment techniques to be identified (4, 14, 15, 16). Although various enrichment methods for glycan moieties have been developed, the lack of unbiased and deep enrichment strategy is still a main limiting factor. In addition, the high heterogeneity of glycans on both N- and O-linked glycosites results in an increased number of glycoproteoforms, which can further dilute glycopeptide signals. In addition, some glycans (*e.g.*, SA) can suppress ionization efficiency of glycopeptides (17). These unique features of glycoproteomics pose more specific challenges in MS analysis than in any other PTM-omics.

Although no single method can address all the issues in glycoproteomics mentioned previously, DIA has been proven powerful and attractive in various experimental setups and is now starting to be applied to the field of glycoproteomics. DIA-based MS can address some of the major problems of DDA-based glycoproteomics and significantly increase the number of identified glycopeptides, especially where glycopeptides represent only a small fraction of total peptide pool. DIA-based approaches can also provide more efficient estimation of glycosylation site occupancy and heterogeneity (18). The benefits of DIA, including broader dynamic range, improved reproducibility, and accurate quantification, provide unique support for efficient PTM analyses. Whereas DIA requires more elaborate data processing strategies to provide sufficient information for a reliable glycan identification and site localization, it is becoming more predominant, and a number of DIA-based studies have been published recently, especially for phosphoproteomics, such as Thesaurus (19) and phosphoDIA (20).

Here we review the applications in DIA-based glycoproteomics that has allowed specific and/or thorough analysis of glycoproteomes. Apart from studies for glycan oxonium ion profiling, which may also include other types of glycosylation, applications of DIA in glycoproteomics are still limited to N-glycoproteomes and GalNAc-type O-glycoproteomes. Hence, in this review, we cover developments and basic principles of DIA strategy and different application aspects in glycoproteomics: (1) DIA on N-linked deglycosylated peptides; (2) DIA on intact N-glycopeptides; (3) DIA on glycan oxonium ions; and (4) Glyco-DIA on GalNAc-type O-glycopeptides (Fig. 1*C*).

## Development and Applications of DIA in Glycoproteomics

### The Development of DIA Strategy

Recent developments in DIA analysis have greatly advanced proteomics. In terms of peptide identification, coverage, reproducibility, accurate quantification, and scalability, DIA significantly outperforms DDA. Numerous developments in DIA strategy were documented (21, 22, 23, 24, 25, 26, 27), extensively compared, and discussed in recent reviews (13, 28, 29). An important achievement in DIA development arrived in 2012 when Gillet *et al.* (25) published the sequential window acquisition of all theoretical mass spectra (SWATH-MS) technology. In SWATH-MS, a Q-time of flight mass spectrometer repeatedly measures fragment ions of the same peptides during elution time while data analysis is based on prior knowledge from spectral libraries. Typically, DIA is performed on mass spectrometers with a quadrupole as the first mass analyzer and a time of flight or Orbitrap as a second mass analyzer.

Since DIA applies much wider precursor isolation windows, multiple precursors are cofragmented simultaneously, and all generated fragments are grouped. Fragment ions' assignment and interpretation of such chimeric MS/MS spectrum is one of the most challenging tasks in the DIA approach. So far, two concepts for the analysis of DIA data could be applied: spectrum-centric and peptide-centric scoring methods (30, 31, 32, 33, 34). Spectrum-centric scoring methods are widely applied in DDA mode where fragment ions are compared and aligned with those generated *in silico* from protein databases to score the most probable peptide sequence. This concept was implemented in the DIA strategy (31, 35), such as the method called DIA-Umpire where DIA MS/MS spectrum is first deconvoluted into multiple pseudo MS/MS spectra based on the correlation between fragment ions and retention time (RT), with each of these spectra comprising fragment ions from individual peptides in the mixture. These spectra can then be searched by traditional DDA spectrum-centric scoring methods. In peptide-centric scoring methods, a predefined peptide of interest is queried against the selected database to align with the best-fit candidate using a set of specific parameters. In software adopting these methods, such as OpenSWATH, Skyline, and Spectronaut (25, 36, 37, 38, 39), spectral libraries are generated from prior DDA identifications using extracted fragment ion chromatograms as identification metrics. Each peptide candidate from the DIA spectrum is scored according to the relative intensity of its fragment ions and accuracy of its RT alignment.

Notably, substantial improvements in informatics for DIA methodology have been achieved with the help of machine learning, especially deep learning technologies. Since peptide-centric approaches still outperform spectrum-centric approaches (13, 40), a major focus of deep learning methods in DIA is to generate spectral libraries *in silico* by predicting RT and peptide tandem mass spectra. Various methods for RT prediction have been made with both machine learning–based techniques and other approaches, such as index-based methods and modeling-based methods (41). Although tools for tandem mass spectrum prediction can be traced back to the early 2000s (42, 43), their performance was often limited. Recently, several models capable of accurate prediction have been trained and developed with different machine learning architectures (44, 45, 46, 47). Remarkably, almost all of them can generate peptide fragments as that correlate well with experimental spectra and have been increasingly implemented in various DIA experiments. Meanwhile, deep learning–based data processing tools developed for DIA data analysis represent a promising future direction (48, 49).

### DIA on N-Linked Deglycosylated Peptides

**Fig. 2:**
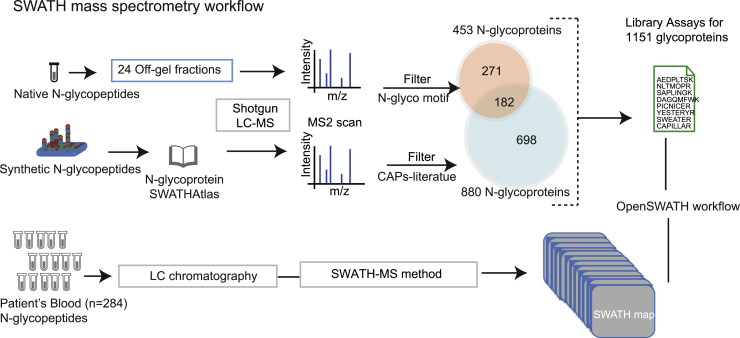
**Schematic representation of the SWATH-MS workflow and SWATH assay library generation from native and synthetic glycopeptides.** Note, all the N-linked glycopeptides were deglycosylated by PNGase F prior to MS analysis. Reprinted from Sajic *et al.* (54) with permission from the author. SWATH-MS, sequential window acquisition of all theoretical mass spectra.

Deglycosylation of N-glycopeptides with enzymes such as peptide-N-glycosidase F (PNGase F) introduces asparagine (Asp) deamidation. Screening for such deamidated peptides enables N-glycosites mapping and is a widely used approach in many N-linked glycoproteomics studies as it can decrease sample complexity and thus lower analytical barriers (50). Analysis of deglycosylated peptides (deglycoproteomics) does not meet the strictest definition of glycoproteomics, but it has largely expanded the known N-glycoproteomes and can efficiently pinpoint N-glycosites (51). As the analyte is essentially nonglycosylated peptides, the capability of deep proteome coverage, accurate label-free quantification, and a high degree of reproducibility make DIA an attractive approach for analysis of deglycosylated glycoproteomes. Liu *et al.* (52) applied a workflow that combined N-glycoproteome enrichment and the SWATH-MS method to quantitatively measure enriched N-linked glycoproteins in human plasma and showed its potential for biomarker discovery. The study systematically compared the performance between SWATH and selected reaction monitoring (SRM), concluding that SWATH resulted in a similar performance in variability, accuracy, and dynamic range with a slightly lower sensitivity but much deeper glycoproteome coverage. Following the same analytical strategy, Liu *et al.* (53) measured N-glycoproteome samples from prostate cancer tissues and isolated potential biomarkers of prostate cancer aggressiveness. Remarkably, considering the completeness of data acquisition and capability of retrospective querying, SWATH-MS permits the simultaneous quantification of numerous deglycosylated peptides, enabling a substantial increase in identification numbers with 1430 N-glycosites per sample. More recently, another study from the same group analyzed N-glycoproteomes covering thousands of deglycosylated peptides from 284 blood samples from patients with five different solid carcinomas and controls (54) (Fig. 2). With the help of OpenSWATH (55), which automates the targeted analysis of DIA data, researchers were also able to conduct even more high-throughput analyses of the N-glycoproteome focusing on deglycosylated peptides. Nigjeh *et al.* (56) adopted a similar analytical pipeline for DIA by applying Orbitrap instruments for the analysis of enriched deglycosylated plasma peptide samples from patients with pancreatic cancer and healthy controls, reporting galectin-3–binding proteins (LGALS3BP) as elevated in the plasma of the patients.

Another possible application of the analysis of deglycosylated peptides concentrates on accurate quantification of macroheterogeneity (glycosylation occupancy or stoichiometry) on individual glycosites. Ideally, to measure macroheterogeneity, glycopeptides and their nonglycosylated counterparts need to be treated unbiased and simultaneously captured. Hence, ideally no glycosylation enrichment should be conducted during sample preparation. Xu *et al.* (57) established a pipeline for site-specific occupancy measurement of various glycosites in wildtype and glycoengineered yeast cell wall and human saliva samples. In this pipeline, instead of complete removal of the glycan moieties by PNGase F, they treated the samples with Endo H, which cleaves high-mannose type N-glycans while leaving behind a single N-acetylglucosamine (GlcNAc) on the Asn residue. They measured eight glycosites by identifying both deglycosylated and unmodified forms. Furthermore, they also calculated the ratio between abundances of glycosite-containing peptides and all detected peptides in corresponding glycoproteins. This ratiometric strategy allowed for increased accuracy of macroheterogeneity measurement on 20 glycosites.

In addition, an advantage of using Endo H instead of PNGase F is based on the fact that the mass difference of 203.08 Da caused by the remaining GlcNAc can lead to the two forms falling into different SWATH windows, resulting in an easier and more reliable data analysis. This strategy was used to further develop a targeted DIA and a pseudo-SRM method (58) designated as SWAT-MS (sequential window acquisition of targeted fragment ions) (59). Unlike the SWATH-MS that acquire all theoretical fragment ions, the SWAT-MS method only isolates selected peptides of interests with 4 *m*/*z* windows. In other words, the SWAT-MS method is a targeted MS/MS approach, but without the need for elaborate optimization of the transitions. That study benchmarked the performance among SRM, SWAT-MS, and SWATH-MS and concluded that SWAT-MS provides higher sensitivity and improved linearity than SWATH-MS. A similar analysis to measure the macroheterogeneity in yeast cell wall samples with the Endo H deglycosylation step was also conducted. As expected, SWAT-MS was able to detect more N-glycosites with higher sensitivity than SWATH-MS. Yang *et al.* (60) reported a strategy for in-depth measurement of N-glycosylation stoichiometry in mammalian cell line samples with either tunicamycin treatment or a temperature shift. Remarkably, macroheterogeneity of a total of 2274 N-glycosites was characterized in this study. To achieve such a higher coverage, in-depth spectral library of deglycosylated peptides enriched with lectins (concanavalin A, wheat germ agglutinin, and *Ricinus communis* agglutinin) and then treated by PNGase F was generated for deglycosylated peptide identification. This library was also converted to a spectral library for the nonglycosylated peptides by changing all relevant fragment ions. In addition, deglycosylated peptides with lectin enrichment and flow-through samples were then analyzed separately with SWATH-MS. While the deglycosylated peptides and their nonglycosylated counterparts were not treated unbiasedly and acquired in the same MS runs, macroheterogeneity was calculated indirectly by the intensities of these two forms from different runs. As demonstrated by various studies, the spectral library is normally the key factor for DIA experiments in glycoproteomics and proteomics in general. Project-specific libraries are naturally more suitable and needed to be generated for each study. Nevertheless, all publicly available spectral libraries can also be used to build targeted MS/MS and/or DIA methods for glycoproteomics including measurement of macroheterogeneity as demonstrated by Poljak *et al.* (61).

### DIA on Intact N-Glycopeptides

The aforementioned DIA strategy for mapping N-glycosites is based on screening of nonglycosylated peptides generated from enzymatically deglycosylated peptides. Under these conditions, standard DIA protocols developed for proteomics could be effectively applied. In the analysis of intact N-glycopeptides, DIA methodology requires certain adjustments and optimizations on LC–MS settings (*e.g.*, *m*/*z* ranges) as well as on data analysis pipelines (*e.g.*, specific and curated glycopeptide spectral libraries). Zacchi and Schulz (62) pioneered DIA-based N-glycoproteomics of intact glycopeptides, revealing defects in mature proteins caused by mutations in the N-glycosylation pathway in various *Saccharomyces cerevisiae* strains. Based on their previously described SWATH-MS protocol for macroheterogeneity quantitation (57), the authors omitted the deglycosylation step in this study and were able to measure microheterogeneity and macroheterogeneity at eight different N-glycosites. Notably, to facilitate automated identification and quantification with SWATH-MS, spectral libraries of the glycopeptides bearing these glycosites were generated using fragment ions from their nonglycosylated counterparts instead of their innate fragment ions. As an alternative strategy, Sanda and Goldman (63) reported a SWATH-MS–based DIA pipeline for the detection of intact IgG glycoforms from human plasma using Y-ions generated under minimal fragmentation of glycopeptides. These manually curated Y-ions with a high yield of up to 60% of precursor intensity were proven to be highly specific to each glycoform (64). With this approach, the authors were able to detect approximately 20 glycoforms in different IgG subclasses and to monitor their changes in patients with liver cirrhosis. In a follow-up study using the same approach, they constructed a spectral library containing 161 glycoforms of 25 peptides from 14 protein groups and were able to detect 10 of 14 glycoproteins without any glycopeptide enrichment, revealing glycosylation changes between cirrhotic patients and healthy controls (65).

**Fig. 3:**
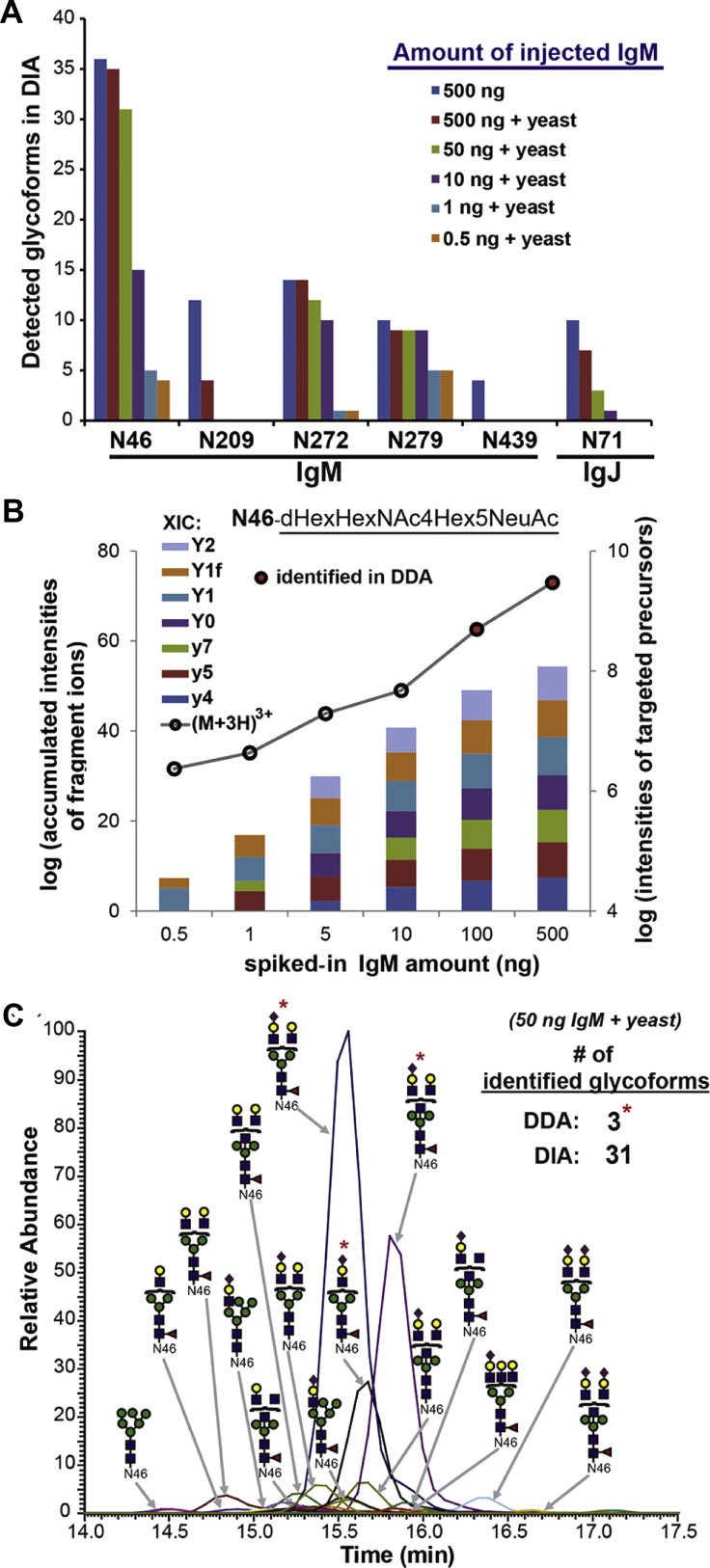
**Target analysis by DIA approach provides better sensitivity than DDA.***A*, the numbers of successfully detected site-specific IgM/IgJ glycoforms with matching MS1 precursor and MS2 fragment-ion chromatograms extracted from DIA data acquired from 500 ng of injected IgM, compared with samples containing decreasing amounts of IgM in yeast lysates. *B*, bar chart of accumulated intensities of various fragment ions from the N46 peptide “YKNNSDISSTR”, carrying the glycan “dHex(1)HexNAc(4)Hex(5)NeuAc(1)”, in yeast lysates containing increasing amount of IgM. *Open circles* connected by *gray line* indicate the XIC peak intensities of that particular N46 glycopeptide precursor in each of the samples analyzed by DIA approach. The *circles* are colored as *red* if this glycoform was also identified by the corresponding DDA analysis. *C*, overlaid precursor XICs of all IgM N46 glycoforms detected by DIA analysis of 50 ng of IgM spiked in yeast lysates. Potential glycan structures were annotated for the more abundant peaks. Glycoforms that were also identified by DDA in the same sample are indicated with an *asterisk*. Among the 31 glycoforms detected by DIA, only the most abundant three were identified by DDA. Reprinted from Pan *et al.* (66), with permission from the author. DDA, data-dependent acquisition; DIA, data-independent acquisition.

Pan *et al.* (66) established a DIA method for site-specific N-glycosylation analysis of six glycosites in IgM (one glycosite from conjunctive IgJ) on a quadrupole-Orbitrap instrument (Fig. 3). Based on a systematic evaluation of the fragmentation behavior of target glycopeptides, the authors built a spectral library/transition containing both glycan Y-ions and peptide fragments, which resulted in a balanced selection of sensitivity and specificity. Meanwhile, as DIA acquires MS/MS information over the full-scan range, DIA raw files can always be reanalyzed postacquisition. This unique feature thus led to potential discovery of unknown and/or undefined modifications on an IgM glycopeptide by analyzing its previously identified glycoforms. Simultaneously and independently, Lin *et al.* (67) developed a workflow showing the wider applicability of this concept in complex matrices and were able to detect 59 glycopeptides without experimental spectral libraries. Large glycan moieties on glycopeptides can also alter *m/z* distributions of precursors compared with nonglycosylated peptides. Hence, full mass ranges and arrangements of windows in DIA method should also be adjusted in DIA-based glycoproteomics studies. Zhou and Schulz (68) developed a SWATH method with optimized variable windows and demonstrated improved characterization of glycopeptides, especially those that bear large glycans.

### DIA on Diagnostic Glyco-Oxonium Ions

In glycoproteomics, oxonium ions are small glycan fragment ions generated during collisional induced dissociation/higher-energy collisional dissociation (HCD) fragmentation (69). Oxonium ions can be originated from glycopeptides with any type of glycosylation, and an unbiased screening using DIA methods for the “oxoniome” can thus provide information on both glycan compositions and help with structure elucidation. Importantly, this type of analysis does not require prior knowledge in terms of glycosylation in samples. To date, two studies have been carried out to profile the oxoniomes. Madsen *et al.* (70) used a DIA method to generate diagnostic oxonium ion profiles for quantitative assessment of IgG glycosylation. Two types of highly abundant oxonium ions, HexNAc (*m/z* 204.09, C_8_H_13_O_5_N_1_) and SA (*m/z* 274.09, NeuAc–H_2_O, C_11_H_15_O_7_N_1_), were monitored using all ion fragmentation in which all precursors in the defined mass range were fragmented without mass filtering (71). With this oxonium ion profiling method, the authors measured the presence or the absence of Fab glycosylation in a therapeutic monoclonal antibody and also compared multiglycosylated biotherapeutics in a high-throughput manner.

This oxonium ion profiling method represents an unbiased approach for a quick differentiation of multiglycosylated biotherapeutics or other glycoprotein samples. Using this approach, Phung *et al.* (72) developed an automated ion library generator designated DIALib and profiled the oxoniomes in cell wall samples from wildtypes of yeast and its nine glycosylation mutants using the SWATH-based DIA approach. Note that, although the initial biosynthesis pathway and early processing steps before the production of Man_8_GlcNAc_2_ glycan are identical in yeast and mammals, yeast tend to produce hypermannosylated glycans by adding additional mannoses (73). Therefore, the spectral library used in this yeast cell wall study consisted of eight most common oxonium ions, which were all related to Hex and/or HexNAc. The authors then summed their signal intensity in each window before normalization to the total oxonium intensity for each strain. With this approach, the authors were able to monitor effects on glycosylation occupancy and glycan structure in mutant strains as well as the overall monosaccharide composition in the glycoproteomes.

This global screening of oxonium ions in glycoproteomics samples is a unique feature of the DIA method. Using DIA oxonium fragments could be screened from almost all precursor ions across the full MS range, and each DIA scan range could be associated with the corresponding RT. Moreover, essentially all the DIA data regardless of whether it is from glycoproteomes can be easily (re)analyzed with this approach. Theoretically, it has the potential to screen the entire glycome limited only by the types of oxonium ions and other diagnostic MS signals (*i.e.*, glycan neutral loss). Hence, we envision that a more sophisticated pipeline for this analysis can be developed and made accessible to the broader proteomics community. Nevertheless, the screening of oxonium ions does not discriminate against the origin of them especially in the case of coeluting glycopeptides or the presence of multiple glycosylation sites at a single peptide backbone. Thus, it cannot provide any information about glycosites or glycoproteins. So far, that approach is also limited to a few types of oxonium ions.

### DIA on O-Glycoproteomics: Glyco-DIA

**Fig. 4:**
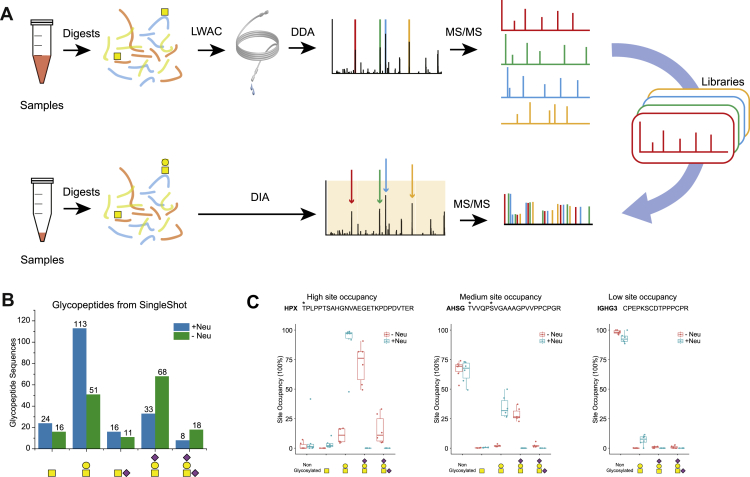
**Overview of the Glyco-DIA strategy.***A*, graphic depiction of the workflow for generation of DIA glycopeptide libraries and the DIA workflow for direct glycoproteomic analysis with Glyco-DIA libraries. A large amount of protein digests is enriched with LWAC and analyzed in DDA mode to build the Glyco-DIA library. Proteomic samples thus can be analyzed directly in DIA mode without glyco enrichment. *B*, numbers of glycopeptides with various structures identified in single-shot analysis. *C*, three examples for three glycosites identified with different glycoforms, representing high, medium, and low glycosite occupancies in all the six serum control samples. Letters with ∗ represent previous identifications in O-glycoproteome database. Reprinted from Ye *et al.* (18), with permission from the author. DDA, data-dependent acquisition; LWAC, lectin weak affinity chromatography.

Although several attempts to apply DIA toward N-glycoproteomics studies have shown great promise of the method, its implementation into the field of O-glycoproteomics is still in its infancy. We proposed a DIA-based strategy for O-glycoproteomics designated as Glyco-DIA to bring forward a high-throughput analytics enabling quantitative O-GalNAc–type glycoproteomics in complex biological samples (18) (Fig. 4). To conduct O-glycoproteomic analysis in DIA mode, high-quality glycopeptide fragmentation spectra are essential. We took advantage of SimpleCell glycoproteomics platform, which can produce homogeneous O-glycans on each glycosite to generate HexNAc (Tn-) peptide spectral libraries. In addition, we also generated Hex-HexNAc (T-) peptide spectral library from wildtype cell lines and human serum samples. The combined Tn/T-peptide libraries contained more than 2000 glycoproteins with more than 11,000 unique glycopeptide sequences, representing the most global human O-glycoproteome (74).

While the lectin weak affinity chromatography–based DDA approach gives a deep coverage of glycosites and glycopeptides, the glyco structures are limited to Tn and T epitopes owing to lectin specificity. It is hard to find a lectin of the same level of specificity and efficiency; for example, for sialylated glycoforms to generate DDA library. As lack of specific and efficient lectins for sialylated glycoforms precluded us from generating DDA library, we developed an *in silico* approach to expand the spectral library with more structures making use of unique features of HCD fragmentation for glycopeptides. By comparing technical replicates of Tn- and T-peptide libraries, we systematically showed that the fragmentation patterns of the peptide of different glycoforms are common and highly reproducible. Therefore, we can simply modify the precursor ion mass in the library and apply parent spectral libraries for other glycan structures. Based on Tn- and T-libraries, we expanded them *in silico* for NeuAc–HexNAc (STn), NeuAc–Hex–HexNAc (ST), and NeuAc2–Hex–HexNAc (diST) epitopes as well as for the nonglycosylated form. This *in silico* approach enables application of DIA strategy for identification of glycopeptides, where DIA libraries cannot be readily generated from real DDA runs directly.

Applying Glyco-DIA method to human serum samples enabled to employ deep and reproducible quantitative analysis of O-glycopeptides with five different glycovariants (Tn, T, STn, ST, and diST) in a single-shot analysis without prior enrichment. Glyco-DIA is expandable and widely applicable to different glycoproteomes and other PTMs, and it may represent the first direct and thorough approach to O-glycoproteomics (Fig. 4).

## Limitations and Further Perspectives

### Detection Sensitivity

Compared with DDA, a key factor leading to the increase of identifications in many DIA experiments is the more efficient use of the ion beam (20). DIA does not necessarily improve the sensitivity to generate more identifications. Instead, the sensitivity can even be hampered by the wide mass windows. Thus, DIA is still not able to measure glycopeptides with low abundance levels that can be detected by SRM/parallel reaction monitoring targeted analysis. Nevertheless, in shotgun analysis, DIA could be an effective alternative for large-scale sensitive quantification of glycopeptides compared with DDA-based approach. This can provide a significant increase in the identification rate of low abundant glycopeptides and potential determination of glycosite occupancy (18). Thereby, further developments in DIA glycoproteomics could bridge the technology with single-cell approaches and enable spatial glycoproteomics discovery in tissues. Wider application of this strategy will expand our knowledge about glycosylation and open a treasure trove of data ready to be mined for biomarker discovery or therapeutic targets in health and disease.

### Site-Specific DIA

Currently, because of fragmentation efficiency of different glycopeptides and scan speed, HCD MS/MS is the only method of choice for DIA. Its major disadvantage in glycoproteomics is the loss of glycan moiety and poor yield of peptide backbone fragments. Nevertheless, under optimized conditions depending on the complexity of the glycan moiety, glycopeptide sequences could still be decoded at a sufficient confidence level. Unfortunately, information about glycosite localization is lost in most cases. Electron transfer dissociation (ETD), as an alternative fragmentation technique capable of mapping glycosite unambiguously, has not been implemented in DIA method yet. Improvements of its efficiency and especially decrease of overall cycle time could empower glycosite localization in DIA-based glycoproteomics. An important question here is “Are there any perspectives to implement ETD in DIA mode?”

It has been demonstrated that positional glycopeptide variants might tend to elute at different RT (18). Pairing HCD and ETD MS/MS spectra *via* the RT could make HCD MS/MS glycopeptide spectra to be site specific. In such cases, ETD DDA MS/MS can provide RT index for unambiguous site localization, whereas HCD MS/MS acquired from the same precursor ions at the same RT would serve as the source of fragment ions for DIA libraries. Therefore, parallel acquisition of the same precursor ions under HCD and ETD fragmentation could make glycopeptide DIA libraries to be partially site specific. Since ETD MS/MS is not directly integrated yet for DIA, further development in this direction could lead to promising applications.

### False Discovery in DIA-Based Glycoproteomics

In general, confident identification of glycopeptides requires to obtain several levels of information: peptide sequence identification, glycan moiety identification, and ideally glycosite localization. A number of studies have been performed to elucidate how to calculate false discovery rate (FDR) for glycopeptides (75, 76, 77, 78). However, this aspect is still under discussion and further development. In DIA-based glycoproteomics, FDR is especially complicated because of the increased complexity of DIA MS/MS spectra. In the case of HCD spectra for glycoproteomics, the same set of fragment ions could be generated for two different glycopeptides common in peptide sequence but different in glyco moiety or even naked (nonglycosylated) peptide. The use of such spectral libraries for DIA analysis may result in a high level of misinterpretations, especially if the glycopeptides coelute and cannot be resolved chromatographically. This problem is particularly important in the case when several glycoforms are present together with nonglycosylated peptide. Because of a high degree of sugar losses under HCD MSMS fragmentation, fragment ions of glycopeptides with different glycoforms will share the same set of ions. Therefore, identifications purely based on MS/MS fragment alignment are not sufficient and could potentially lead to misinterpretation, where peptide sequence is deciphered correctly but PTM information is wrong. Accurate alignment of the precursor ions from the library and full MS scans could partially help to improve FDR control (18), but more study is still required in this field.

### Machine Learning in DIA-Based Glycoproteomics

As mentioned previously, several machine learning–based approaches have been applied recently to predict the fragmentation patterns of MS/MS spectra and provided considerable performance in DIA-based proteomics studies. This could be an even more attractive strategy for glycoproteomics. Although the spectrum-centric methods can be theoretically applied to glycoproteomics, the performance will be limited and hampered by the complexity as well as the incompleteness of fragment ions from glycopeptides. Interpretation of DIA glycoproteomics data still relies on spectrum-centric approach with spectral libraries, which are costly and require specific expertise to build. Even so, using experimental libraries is sometimes troublesome and because of poor coverage of targets of interest. Efficient machine learning–based tools can thus be particularly useful for glycoproteomics. Unfortunately, because of the vast difference of fragmentation patterns between glycopeptides and peptides, such models and architectures currently do not fulfill the requirements of glycoproteomics and cannot be applied directly. Besides, generation of specific models for glycopeptides requests redesign of the architectures and considerable amounts of high-quality experimental spectra, which can be difficult to build. Nevertheless, we envision that further developments in both the areas, machine-learning, especially deep-learning techniques, and glycoproteomic methodology, will empower DIA for its application in glycoproteomics.

## Conflict of interest

The authors declare no competing interests.
